# Gut microbiota metabolites in autistic children: An epigenetic perspective

**DOI:** 10.1016/j.heliyon.2021.e06105

**Published:** 2021-01-29

**Authors:** Hussein Sabit, Huseyin Tombuloglu, Suriya Rehman, Noor B. Almandil, Emre Cevik, Shaimaa Abdel-Ghany, Sanaa Rashwan, Mustafa Fatih Abasiyanik, Mary Miu Yee Waye

**Affiliations:** a Department of Genetics, Institute for Research and Medical Consultations (IRMC), Imam Abdulrahman Bin Faisal University, P. O. Box 1982, Dammam, 31441 Saudi Arabia; b Department of Epidemic Diseases, Institute for Research and Medical Consultations (IRMC), Imam Abdulrahman Bin Faisal University, P. O. Box 1982, Dammam, 31441 Saudi Arabia; c Department of Clinical Pharmacy Research, Institute for Research and Medical Consultation (IRMC), Imam Abdulrahman Bin Faisal University, P. O. Box 1982, Dammam, 31441 Saudi Arabia; d Department of Environmental Biotechnology, College of Biotechnology, Misr University for Science and Technology, P. O. Box 77, Giza, Egypt; e Pediatrics Department, Madinat Zayed Hospital, SEHA, Abu Dhabi, United Arab Emirates; f Pritzker School of Molecular Engineering, University of Chicago, Chicago, IL, 60637, USA; g Institute for Genomics and Systems Biology, University of Chicago, Chicago, IL, 60637, USA; h The Nethersole School of Nursing, The Croucher Laboratory for Human Genomics, The Chinese University of Hong Kong, Shatin, N.T. Hong Kong

**Keywords:** ASD, Autism, Gut, Microbiota, Metabolites, Epigenetics

## Abstract

Gut microbiota has become an issue of great importance recently due to its major role in autism spectrum disorder (ASD). Over the past three decades, there has been a sustained research activity focused to explain the actual mechanism by which gut microbiota triggers/develops autism. Several genetic and epigenetic factors are involved in this disorder, with epigenetics being the most active area of research. Although the constant investigation and advancements, epigenetic implications in ASD still need a deeper functional/causal analysis. In this review, we describe the major gut microbiota metabolites and how they induce epigenetic changes in ASD along with interactions through the gut-brain axis.

## 1 Introduction

**Figure 1:**
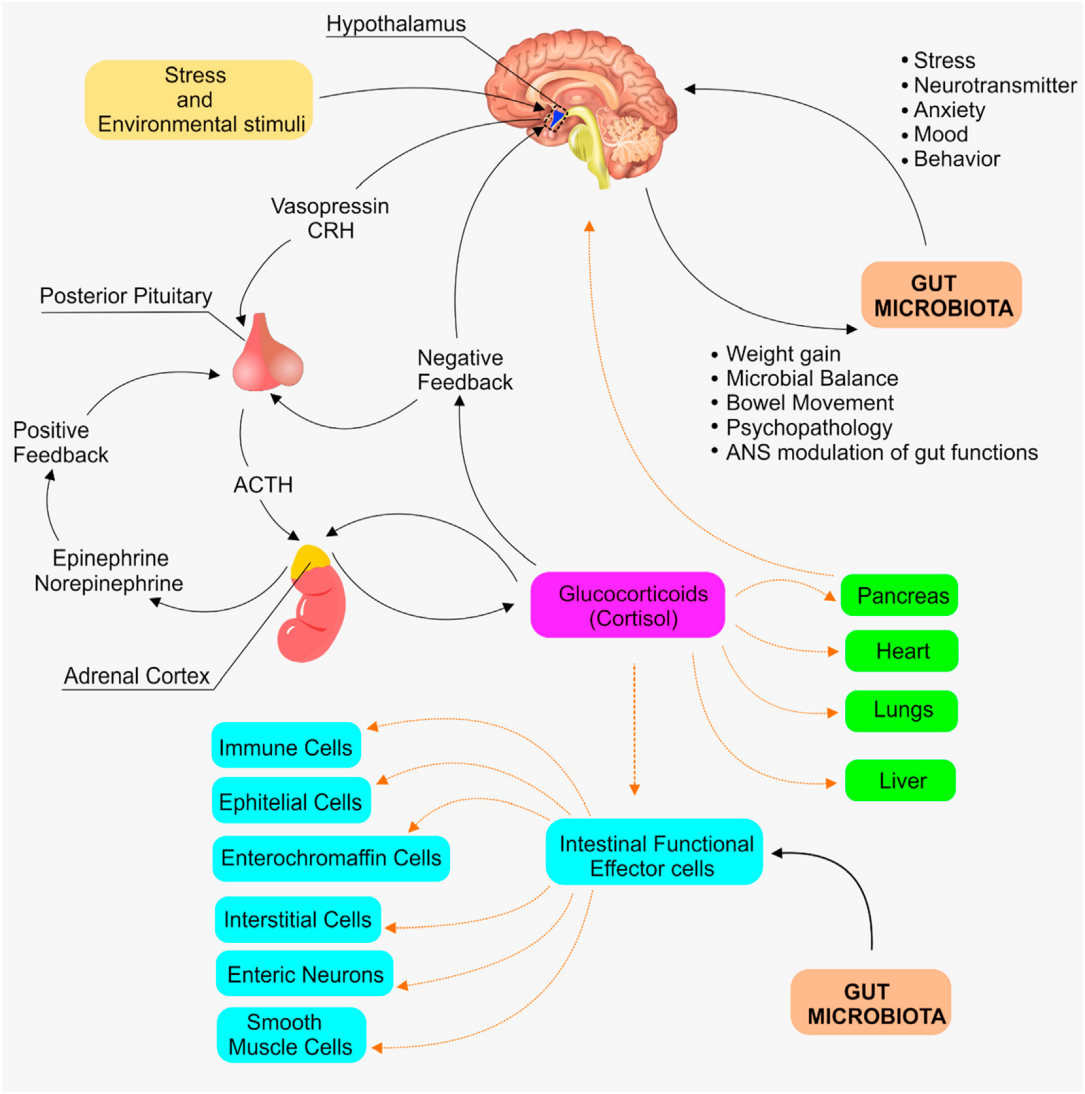
The interaction between gut microbiota and brain via the gut-brain axis. The hypothalamus produces corticosteroid releasing hormones which act on the intestinal functional effector cells where gut microbiota also paly. Via the gut-brain direction, gut microbiota can stimulate changes in stress, mode, anxiety, neurotransmitter, while brain can cause certain changes such as weight gain, microbial balance, bowel movement, psychopathology, and modulation of the guts by ANS. CRH: corticosteroid releasing hormone, ACTH: Adrenocorticotropic hormone, and ANS: Autonomous nervous system.

Autism spectrum disorder (ASD) is a group of neurodevelopmental conditions, characterized by incessant deficits in social interactions and communication, restricted interests, reciprocal social interaction and repetitive behaviors. Prevalence of ASD is estimated to be 14.6 per 1000 in children within eight years of age, having a male to a female ratio of 3:1 [1]. ASD represent a persistent burden not only for autistic persons, but also for the entire society. Previous research has established that genetic and epigenetic factors play a crucial role as underlying factors for ASD. Likewise, studies over the past two decades have provided important information on the role of gut microbiota (GMB) in the severity of ASD. GMB is the collection of microorganisms, with greater abundance of bacteria that reside mostly on the external and internal surfaces of the gastrointestinal tract, skin, and oral mucosa [2, 3]. Recent corrected estimates indicated that human body contains nearly 38 trillion bacterial cells, with the vast majority of them residing in colon [4]. GMB communicates with brain *via* microbiome-gut-brain axis; by which GMB modulate brain development, immunity, and metabolic homeostasis. Disrupting this communication can result in a variety of conditions, including ASD [5, 6]. The gut-brain axis could be affected by the microbiome through different routes:

1. The dendritic cells in the gut which collect metabolites from the microbiome transfer them *via* exosomes into T cells through the lymphatic system, throughout the body.
2. The metabolites could cross the blood brain barrier and affect receptors in the neurons (*e.g. via* the T-like receptors), thus affecting signaling of the brain or developmental process such as pruning of neurons during critical periods of brain development.
3. Microbiome can regulate several key neurotransmitters such as gamma amino butyric acid (GABA), glutamate, serotonin, dopamine, which could in turn lead to emotional and behavioral changes.
4. Interaction between microbiome components or their metabolites could lead to secretion of cytokines, which in turn affect the immune function of the body and activate the hypothalamus-pituitary axis (HPA) *e.g.,* release of vasopressin and corticotrophin-releasing hormone (CRH) that signals for the release of adrenocorticotropic hormone (ACTH) and cortisol.
5. Increase permeability of the intestinal mucosa, leading to transfer of gut components (*e.g.* lipopolysaccharides (LPS) and pro-inflammatory cytokines) or metabolites of the microbiome into the circulatory system, thus affecting the HPA (Figure 1).

In this review, we describe the role of GMB in the modulation of physiological balance, brain function, and behavioral aspects in autistic individuals.

## 2 Autism spectrum disorder

ASD is a group of heterogeneous neurodevelopmental behavioral disorder, which begins in early childhood and tends to persist into adolescence and adulthood of the affected individual. ASD alters the way persons respond and interact with others, and the learning abilities. It was estimated that 1 in 160 children develops ASD during the early 10 years of their life [7], without reliable blood-based diagnostic tool or specific pharmacological treatments [8, 9]. The main etiology of ASD is largely unknown although major genetic abnormalities (*e.g*. Fragile X syndrome, chromosomal abnormalities, copy number variations, incomplete penetrance and variable expression, and monogenic syndromes) could be identified as plausible causes in some cases [10]. These genetic factors are identified in only 25–30% of patients with ASD whose etiology starts during gestation, where maternal diet might affect brain regions correlated with cognition of social interaction. On the other hand, there has been a large array of evidence in the last few decades that highlighted the role of GMB in predisposing children to ASD. This has garnered worldwide attention in the past decade due to deep understanding of the molecular mechanism by which GMB cause ASD. The proposed general mechanism is *via* gut-brain axis, although detailed pathway still to be elucidated.

## 3 Gut microbiota

GMB refers to the entire plethora of microorganisms residing inside the human intestinal tract, which comprises not only bacteria, but also archaea, fungi, protozoa, and viruses. This microbial community lives in symbiosis with the host [11]. The term microbiome refers to the complete set of genomes of this microbial community, which is manifold in number than the genes existing in human genome [12]. Bacteria tremendously outnumber eukaryotic and archaeal microorganisms in the human microbiome by 2–3 orders of magnitude [13] so the term “gut microbiota” refers mainly to bacteria due to its dominance over the other microorganisms. There has been an increased recognition for more attention towards the GMB for its potential benefits to human health, as previously reviewed [14, 15]. In a study, GMB comprises 3.8 × 10^13^ bacterial cells compared to 3.0 × 10^13^ human cells in a 70 kg reference man [16]. Recent years have seen a rise in the number of publications that indicate GMB interacts with the central nervous system through vagal and endocrine pathways which is referred as the gut-brain axis. Nevertheless, the molecular mechanism(s) of this interaction is yet to be explained. These finding implies a role of GMB in human behavior and mental health [17], *via* direct or indirect influence on the host immune system and the metabolism [18].

Although GMB shows enormous diversity among healthy individual, some bacteria dominate the entire microbial community, such as the members of *Firmicutes* and *Bacteroidetes* phyla [19]. Meanwhile, Strati *et al.* [20], observed an increase in the *Firmicutes/Bacteroidetes* ratio in autistic patients and they attributed this increase to the reduction in the relative abundance of the *Bacteroidetes*. Moreover, they also noticed a decrease in the abundance of *Alistipes, Bilophila, Dialister, Parabacteroides*, and *Veillonella* and an increase in *Collinsella*, *Corynebacterium, Dorea*, and *Lactobacillus*. Given the strong association between gut microbiota and brain, it is important to understand the molecular role that GMB plays in brain development and functioning. For this goal, recent evidence indicated a bidirectional and perhaps multidimensional connection between the GMB and the brain. This relationship utilizes well-known neurotransmitters along with small peptide molecules, which are yet to be identified [21].

There exists a very extensive literature in the involvement of GMB in ASD, and several studies indicated the presence of altered GMB [22] and oral microbiota [23] in autistic individuals. For example, recent work investigated the oral microbiota in ASD patients and found that *Rothia* species were more prevalent in children with ASD, compared with typically developing children. In another study, Kushak *et al.* [24], reported no significant difference with regard to the oral microbiota between autistic children and healthy subjects. On the other hand, the typically developing children exhibited an increase in the abundance of *Megasphaera, Moraxella, Neisseria,* and *Gemella,* compared with ASD individual [25]. However, there is little accord on the type of bacteria that are similarly altered among the studied autistic individuals. In addition, little is known about the role of microorganisms other than bacteria in ASD, and this is still an area of active research.

In a recent study, the bacterial populations in autistic and non-autistic individuals were compared and the ASD patients showed a significant reduction in the percentages of *Firmicutes* and a significant increase in the percentage of *Acidobacteria* in feces compared to healthy-children (*p* < 0.05) [26]. The dominant bacteria in the ASD group were *Megamonas*, *Megasphaera*, and *Barnesiella* while the dominant bacteria were *Eubacterium, Ezakiella*, and *Streptococcus* in the healthy control group. However, Nogay *et al.* [27], indicated that the published data concerning the role of GMB in gastrointestinal and behavioral problems with gut microbiota in autistic individuals were very limited and contradictory. This contradiction might be attributed to differences in age of enrolled subjects, sample size, type of sample (fecal samples or GI biopsies), and geographical distribution [28]. Wang *et al.*, investigated the gut microbiota in 92 children with ASD [29]. Using shotgun metagenomic sequencing and liquid chromatography-mass spectrometry (LC-MS), they identified the gut metabolites associated with altered gut microbiota composition. Their analysis indicated an altered glutamate metabolite in ASD children associated with a decline in 2-keto-glutaramic acid and an abundance of microbiota associated with glutamate metabolism. This disturbance in glutamate metabolism was associated with lower levels of *Bacteroides vulgatus* and higher levels of *Eggerthella lenta* and *Clostridium botulinum*. Recent estimates reported that individual human gut is colonized by 500 to 1,000 bacterial species. Collectively, the total number of bacterial species colonizing in human guts is about 35,000 species, and the number is continually increasing [30]. Wang *et al.* [31], identified gut microbiota-associated epitopes (MEs) by shotgun metagenomic DNA sequencing of fecal samples, obtained from 43 ASD children (19 with and 24 without GI involvement) and 31 sex- and age-matched typically developing (TD) children. They demonstrated an abnormal MEs composition in the ASD group, which was associated with abnormal gut IgA levels. This may suggest an abnormal intestinal immunity in ASD children. This indicates that ASD patients with GI symptoms show behavioral indications, such as anxiety, self-injury, and aggression [32]. Considering these recent works in the field of GMB, and taking in consideration some contradictions, it is becoming extremely important to deeply investigate the microbial community in autistic children and validate the proposed gut-GMB dysbiosis.

### 3.1 Dysbiosis of gut microbiota

The normal symbiotic relationship between gut microorganisms and the gastrointestinal tract, has substantial impact on the gut homeostasis [33]. Disruption of gut microbial balance is referred to as dysbiosis. Dysbiosis (*aka*. dysbacteriosis) refers to an imbalance in the microbial community of the human body. In this case, pathogenic bacteria can outnumber the beneficial ones, leading to complicated disorders in the host gastrointestinal tract [34], where small intestinal bacterial overgrowth (SIBO) is the result [35, 36]. Nevertheless, the actual role of fungi dysbiosis in ASD needs more elucidating studies with larger samples. Various researches highlighted the association of dysbiosis with pathogenesis of either intestinal disorders, such as irritable bowel syndrome (IBS) and coeliac disease, or extra-intestinal disorders like, allergy, asthma, cardiovascular disease, obesity, and autism [37]. More recent studies reviewed by Afroz *et al.* [38], has indicated that the excessive salt consumption can disrupt the gut microbiota resulting in dysbiosis, which may induce several pro-inflammatory pathways, including differentiation of T helper-17 and regulatory T cells, downregulation of M2 regulatory macrophages, and over activation of M1 pro-inflammatory macrophages.

Connolly *et al.* [39], reported that maternal high-fat diet during pregnancy can cause microbial dysbiosis, which may increase the probability that the neonate may undergo ASD. Studies also have indicated that breast-feeding decreases the risk of developing ASD, while formula-feeding increases the abundance of *Clostridium difficile* in the infant's gut, which is associated with higher risk of ASD [40, 41, 42].

Some antibiotics are known to enhance gut microbiota, which results in eubiosis, while others may have harmful effects and hence cause dysbiosis. This dysbiosis might lead to ASD [43, 44]. Thus, antibiotics can alter the GMB, either positively or adversely. As autistic children are more prone to infections, they receive higher doses of antibiotics, and this may lead to increased abundance of *Desulfovibrio* species. This bacterial species can stimulate the pathogenesis of ASD *via* producing some lipopolysaccharides, that function as virulent factors [45].

The correlation between ASD and specific species of bacteria has been highlighted in various previous studies. Xu *et al.* [46], showed that children with ASD had low percentage of *Bifidobacterium, Bacteroides, Enterococcus*, and *Escherichia coli*, and high percentage of *Faecalibacterium*. Meanwhile, characterization of fecal samples from ASD patients, a lower level of *Firmicutes* and a higher level of *Bacteroidetes* have been reported [47]. *Bacteroidetes* are short-chain fatty acid (SFA)-producing bacteria that biosynthesize propionic acid, among others, which may impact the CNS and autism behavior *via* modulating the gut-brain axis.

The genus *Clostridium* (belonging to *Firmicutes* phylum) is documented to be overgrown in autistic children [48, 49, 50]. Recent studies have indicated higher occurrence of *Clostridium histolyticum* and *Clostridium perfringens* in stool samples of autistic children [48, 49, 51]. The role of *Clostridium* in ASD was supported by a study in which autistic children were subjected to an oral course of vancomycin against *Clostridium*. The result was a substantial progression of gastrointestinal and neuro-behavioral [52]. Cumulative research findings pointed to a range of metabolites produced by *Clostridiaceae*, which include indole derivate phenols and p-cresol [53, 54, 55]. The toxin-producing *Clostridia* are glyphosate-resistant bacteria, and the exposure of children to this environmental pesticide may increase the abundance of *Clostridia* in their guts, which can lead to ASD progression [56].

Furthermore, dysbiosis of GMB have been reported in ASD animal models and also in children with pervasive developmental disorder-not otherwise specified (PDD-NOS) [18, 57, 58]. Other drugs may contribute in the initiation/progression of ASD. When administered to pregnant mice as an antiepileptic drug, valproic acid induced an autism-like disorder and altered the ratios of *Firmicutes* and *Bacteroidetes* in the offspring [59].

### 3.2 Gut microbiome metabolites

In recent years there has been an increased interest in exploring the role of GMB metabolites, particularly, short-chain fatty acids (SCFAs) in the pathogenesis of ASD. SCFAs are mainly consisting of butyrate (BT), acetic acid (AA), valeric acid (VA), and propionic acid (PPA). The proposed action of these SCFAs is the modification of mitochondrial function in terms of the citric acid cycle and carnitine metabolism, or *via* epigenetically modulating the ASD-related genes [60].

PPA is one of the major SCFAs that are produced by GMB (such as *Clostridia*, *Bacteroides*, *Desulfovibrio*) in children with autism. Getachew *et al.* [61], reported that PPA disturbs GI function in a manner of abnormalities presented in the individuals with ASD. PPA can cause reversible behavioral, neuro-inflammatory, metabolic, and epigenetic changes that resemble those developed in ASD animal model.

BT is one of the most important SCFAs that is primarily produced by the GMB. It positively modulates mitochondrial function and has been proposed as a neuro-protectant. The main functions of BT are to inhibit histone deacetylases (HDAC), regulate the blood-brain barrier (BBB), and suppress intestinal pro-inflammatory macrophage function. For that reason, BT-producing bacterial taxa are less abundant in autistic individuals [62]. To study the role of BT in modulating mitochondrial functions in autistic individuals, Rose *et al.* [63], developed a lymphoblastoid cell line model for ASD, either displaying mitochondrial dysfunction or normal mitochondrial function. They reported that the BT produced by GMB modulated mitochondrial activity, and this modulation was concentration- and microenvironment redox state-dependent.

Although several researches indicated the alteration of GMB and hence the fecal SCFAs in children with autism [64], there is no consensus on the relationship between GMB and fecal SCFAs in autistic children. ASD seems to be a result of interaction between GMB metabolites and genetic factors. GMB composition is affected mainly by the individual lifestyle and surrounding environment. Recent studies have indicated that female exposure to certain environmental factors during pregnancy increases the chance of ASD in offspring, although the molecular mechanism still to be identified [65].

Mouse valproic acid (VPA) models are very useful in outlining the molecular mechanisms underlying ASD behavior and to screen for new therapeutic options [66]. To test the effect of VPA, as an environmental factor, on the development of ASD, Mahmood *et al.* [67], established a mouse model by exposing embryos at day 13, to different concentrations of VPA. A dysregulation of synaptic structure in cortical neurons of the exposed mice was observed. They concluded that exposure to VPA was correlated with a dysregulation in the expression of PTEN (phosphatase and tensin homolog) protein with ASD-like behavioral, and this may be a potential mechanism for VPA-induced ASD. Similar results were reported by Ha *et al.* [68], where they induced ASD in mice using VPA, and treated the affected mice with human adipose-derived stem cells (hASCs) to control the autistic behavior in treated VPA-exposed mice. hASCs treatment resulted in a downregulation of PTEN and restoration of the normal expression level of IL-10 and VEGF in the treated mice. Meanwhile, Wang *et al.* [69], generated also a mouse model for ASD using a single dose of VPA prenatally to pregnant mice. Their results indicated that the exposure to VPA induced a noticeable retardation in the juveniles’ motor reflexes and impaired learning in adult mice. It is generally agreed that maternal use of VPA during pregnancy increases the risk of ASD in children. Therefore, women who have potential for childbearing should take much care, regarding the risk/benefit of using VPA [70].

Higher urine concentrations of 3-(3-hydroxyphenyl)-3-hydroxypropionic acid (HPHPA) were detected in ASD patients suffering from *Clostridium difficile* infections-associated with recurrent diarrhea [71]. Furthermore, Xiong *et al.* [72], also detected HPHPA along with 3-hydroxyphenylacetic acid (3-HPA) and 3-hydroxyhippuric acid (3-HHA) at significantly high concentrations in samples of Chinese children with autism. Further analysis revealed that these compounds were metabolites of gut *Clostridium* species. Increased levels of HPHPA was also identified in a sample of Italian children with ASD [73]. However, oral vancomycin treatment was found to reverse the increased levels of HPHPA, along with two associated metabolites; 3-HPA and 3-HHA [74].

P-cresol, a byproduct of *Clostridium* species, was also detected at significantly (*p* < 0.01) elevated levels in urine samples of autistic children, younger than eight years of age [75]. Altieri *et al.* [75], also reported that high level of urine p-cresol was associated with ASD-like repetitive behaviors.

4-ethylphenyl sulfate (4-EPS), an aryl sulfate that is 4-ethylphenol, is a uremic toxin and a GMB metabolite. 4-EPS not only imparts ASD-like behavior, but also shows several anxiety-like symptoms in normal mice. 4-EPS is not detected in germ-free mice, indicating its origin as a GMB metabolite. Specifically, it is a byproduct of *Lanchnospiraceae* family. 4-EPS was detected at higher levels in offsprings of maternal immune activation (MIA), which was demonstrated in a mouse model that exhibits ASD features. However, low serum levels of 4-EPS were reported in MIA model of ASD. Furthermore, mice treated with a potassium salt of 4-EPS exhibited ASD-like behavior. Treatment with *Bacteriodes fragilis*, a probiotic bacterium, reduced the concentration of 4-EPS in an MIA model of ASD [76]. Being present at detectable concentration in urine of ASD children, it is proposed to be a biomarker for the disease [77].

## 4 Epigenetics of autism spectrum disorder

There is increasing evidence to suggest that environmental and epigenetic factors together play a greater role in the etiology of ASD than previously believed [78]. Different epigenetic mechanisms have been proposed for initiation and progression of ASD. These mechanisms include DNA methylation, histone modifications, miRNA, among others.

### 4.1 DNA methylation

DNA methylation is an epigenetic mechanism for gene expression regulation in response to environmental factors without alterations in DNA sequence [79]. Over the past three decades, there has been a sustained research activity devoted to correlate methylation patterns with ASD, as a result of advancements in next-generation sequencing technologies. Numerous research studies have suggested that ASD is originated in early developmental stages due to an epigenetic delay in the route of regular DNA methylation states throughout the early stages of development. There is a high level of evidence supporting the notion that ASD is either triggered or developed *via* epigenetic mechanisms, however, more elaborations are needed before one can associate specific gene (such as *MECP2*) with the pathogenesis of ASD [80]. However, intensive work has been carried to highlight some genes that are associated with ASD. Among the genes that could be considered as risk factors for ASD is *MTHFR*, which encodes methylenetetrahydrofolate reductase-the key enzyme in the formation of 5-methyl-tetrahydrofolate. Different polymorphisms of this gene such as 677CT/1298AC was found to be associated with ASD (*p* = 0.0207) [81, 82, 83]. Other studies indicated other polymorphisms such as rs1801133 [84], and the discrepancies between these studies is attributed to the inconsistency of the dietary composition in different groups involved in major studies.

Postmortem brain sections of autistic individuals offer a reliable and unique source of data in terms of differential methylation in different brain areas. Numerous genome-scale studies revealed multiple alterations in DNA methylation in the postmortem brains of ASD individuals [85]. Ladd-Acosta *et al.* [78], identified the methylome in brain sections (dorsolateral prefrontal cortex, temporal cortex, and cerebellum) of postmortem tissue from 19 autism cases and 21 unrelated control subjects. They measured 485,000 CpG loci and identified four genome-wide significant differentially methylated regions (DMRs). These DMRs indicate a frequently changed methylation loci in ASD. Corley *et al.* [86], reported a DNA methylation defect in the sub ventricular zone of the lateral ventricles from postmortem brain of 17 autistic individuals and 17 age- and gender-matched normal TD individuals. This methylation defect modified the chromatin structure of genes involved in neurodevelopment, which are, in turn, associated with atypical precursor messenger RNA splicing events of ASD-relevant genes.

A preliminary evidence on the methylation of *HTR2A* in autistic individuals was reported by Hranilovic *et al.* [87], where they found a higher methylation level in autistic individuals with rs6311 AG genotype compared to that of the controls. Meanwhile, Elhawary *et al.* [88], found that heterozygosity contributed to the etiology of ASD for rs3813034 and rs6318SNPs (89%, *p* = 0.005 and 56%, *p* = 0.03, respectively). They indicated also that rs7997012 and rs6265A variant alleles were significantly associated with ASD (*p* = 0.005 and *p* = 0.003, respectively).

An extended epigenome-wide array DNA methylation (DNAm) study was conducted on 968 autistic children. The study analyzed 485,512 CpG sites using the Illumina 450K Beadchip. In that study, Andrews *et al.* [89], compared the top EWAS results (from blood) with those analyzed before from brain samples. However, no significant differences in the DNAm between autistic children and control subjects in the sample size used at a threshold of *p* < 1.12 × 10^− 7^. This might be due to an interaction between ASD polygenic risk score which was found to be associated with DNA methylation increases near a robust ASD signal [90].

Recently, 15 epigenetically modified genes (*EN2, UBE3A, NLGN3, OXTR, MECP2, BCL2, GABA, AFF2, SLC6A4, RORA, NRXN1, AUTS2, RELN, and SHANK3*) related to ASD have been identified (Table 1).

**Table 1:** Examples of epigenetically regulated genes/proteins associated with ASD.

Gene/protein	Function in ASD	Chr. Location	Ref.
*En2*	*Engrailed-2* gene (*En2*) is essential for the topographic mapping of axons in both the tectum and cerebellum	7q36	[91, 92]
*Shank3*	*Shank3* interacts with postsynaptic density proteins. It binds to neuroligins, and then with neurexins to form a functional complex at glutamatergic synapses	22q13	[93]
*RELN*	The Reelin protein is a ligand for LDL-superfamily receptors ApoER2 and VLDLR	7q22	[94]
*RORA*	Retinoic acid-related orphan receptor-alpha (RORA) controls the transcription aromatase, which is very low expressed in the frontal cortex of autistic individuals	15q22.2	[95]
*NLGN3*	*NLGN3* encodes a member of the neuroligin family of neuronal cell surface proteins. Neuroligins may act as splice site-specific ligands for beta-neurexins, and mutations in this gene may be associated with ASD	Xq13.1	[96]
*UBE3A*	*Ubiquitin Protein Ligase E3A* (*UBE3A*) encodes E6AP, a protein that is expressed in a paternally imprinted manner in the brain of patients with ASD	15q11.2	[97]
*BCL2*	*BCL2* is a member of the Bcl-2 family of regulator proteins that regulate apoptosis. It is downregulated in ASD	18q21.33	[98]
*OXTR*	OXTR has important role in the regulation of affiliative behavior and social bonding in humans. It plays a role in ASD, although this role is not well-identified	3p25.3	[99]
*SLC6A4*	It is the paternal version of 5-HTTLPR coupled with prenatal stress. It may significantly affect the risk for offspring to develop ASD	17q11.2	[100]
*GABA*	Gamma-amino butyric acid (GABA) attaches to GABAA receptors to facilitate chloride ions flow across the cell membrane. Its main role is to prevent the brain from being overloaded with too many signals	α1on 5q34-35, α2 and β on 4p12-13, and α3 on Xq28	[101]
*NRXN1*	Neurexins (*NRXN1*) function as cell adhesion molecules and receptors. It is a cell surface receptor that binds neuroligins to form a Ca-dependent neurexin/neuroligin complex at the CNS synapses	2p16.3	[102]
*AUTS2*	The functional role of AUTS2 is not well known, although some studies have identified a putative role in transcriptional regulation during neuronal development.	7q11.22	[103]
*AFF2*	Deletions in this gene result is present with autism	Xq28	[104]
*MECP2*	Methyl-CpG-binding protein-2 (MeCP2) is a regulator for neural development, where LoF or GoF can cause severe neurodevelopmental disorders, including ASD	Xq28	[105, 106]
*SOX7*	Polygenic scores related to two CpG sites located proximal to a robust GWAS signal for ASD	8p23.1	[107]

Liang *et al.* [108], analyzed 5 ASD-discordant monozygotic twins and found 2,397 differentially methylated genes. Among these genes, the methylation of *SH2B1* gene was further studied. A significant DNA methylation difference in ASD-discordant monozygotic twins than ASD-concordant monozygotic twins was observed. These results indicate the altered methylation pattern of *SH2B1* in ASD [78, 86, 109].

MeCP2 is involved in chromatin remodeling and serves as a reader group of proteins. A recent study reported that MeCP2 regulates splicing of specific mRNA *via* crosstalk with 5hmC and modulation of histone tags. Increased MeCP2 interaction with *Reelin* and *glutamate decarboxylase 2* (*GAD2*) promoters was found in some autistic patients, and this may cause downregulation of *GAD2* [110].

### 4.2 Histone modifications

There is a lack of systematic investigation of the association between ASD and histone and chromatin remodeling. Several studies have indicated that dysregulation of various components of the epigenetic machinery can cause cognitive discrepancies at the behavioral level, proposing that accurate epigenetic control is essential for the fundamental functions of brain. Histone modification is among several epigenetic mechanisms that might be involved in the pathogenesis of ASD [111]. Duffney *et al.* [112], reported that out of 215 ASD-related genes, 42 genes were involved in the epigenetic regulation of gene expression, where those genes encode histone- or DNA-modifying proteins. Sun *et al.* [109], analyzed the levels of H3K27ac in 257 postmortem samples extracted from ASD and matched control brains. They found that nearly 68% of both syndromic and idiopathic ASD shared acetylome profile in 5,000 regulatory elements in the prefrontal and temporal cortex. Furthermore, they observed common epimutations in ASD-related genes. Furthermore, they took the next step by identifying 20 genes with histone marks associated with ASD in the brain frontal cortex [113].

*A major* study was conducted by Kim *et al.* [114], demonstrated that non-coding *de novo* mutations participate in the pathogenesis of ASD *via* chromatin interactions. They also found that noncoding *de novo* mutations that affect chromatin interactions exhibited transcriptional dysregulation implicated in ASD risks. Furthermore, non-coding and coding *de novo* mutations were involved in the low IQ of autistic individuals. Their work has proposed five gene (*CTNNA2*, *GRB10*, *IKZF1*, *PDE3B,* and *BACE1)* that were not linked previously to ASD.

### 4.3 Role of miRNAs in autism spectrum disorder

miRNAs are small (18–24 nucleotides) non-coding RNA molecules, which is transcribed in a tissue-specific manner to regulate their target genes [115, 116]. It plays crucial roles in various cellular processes including cell survival, apoptosis, aging, differentiation, carcinogenesis, and metastasis [117]. miRNAs transcribed from various regions in the genome either intragenic or intergenic loci within regions referred to as genome desert [118]. The main function of miRNAs is to post-transcriptionally regulate gene expression, either the gene from which it originates or remotes genes which might be located on different chromosome [119].

In the last decade, various studies have determined the role of miRNA in the etiology of ASD, and several studies indicated an altered expression of different miRNAs in autistic individuals. For instance, high throughput miRNA microarray studies indicated that miRNAs have differential expression profiles in autistic individuals. One of these studies conducted by Huang *et al.* [120], found that the differentially expressed miR34b was prevailed in a higher percentage of male autistic patients.

**Figure 2:**
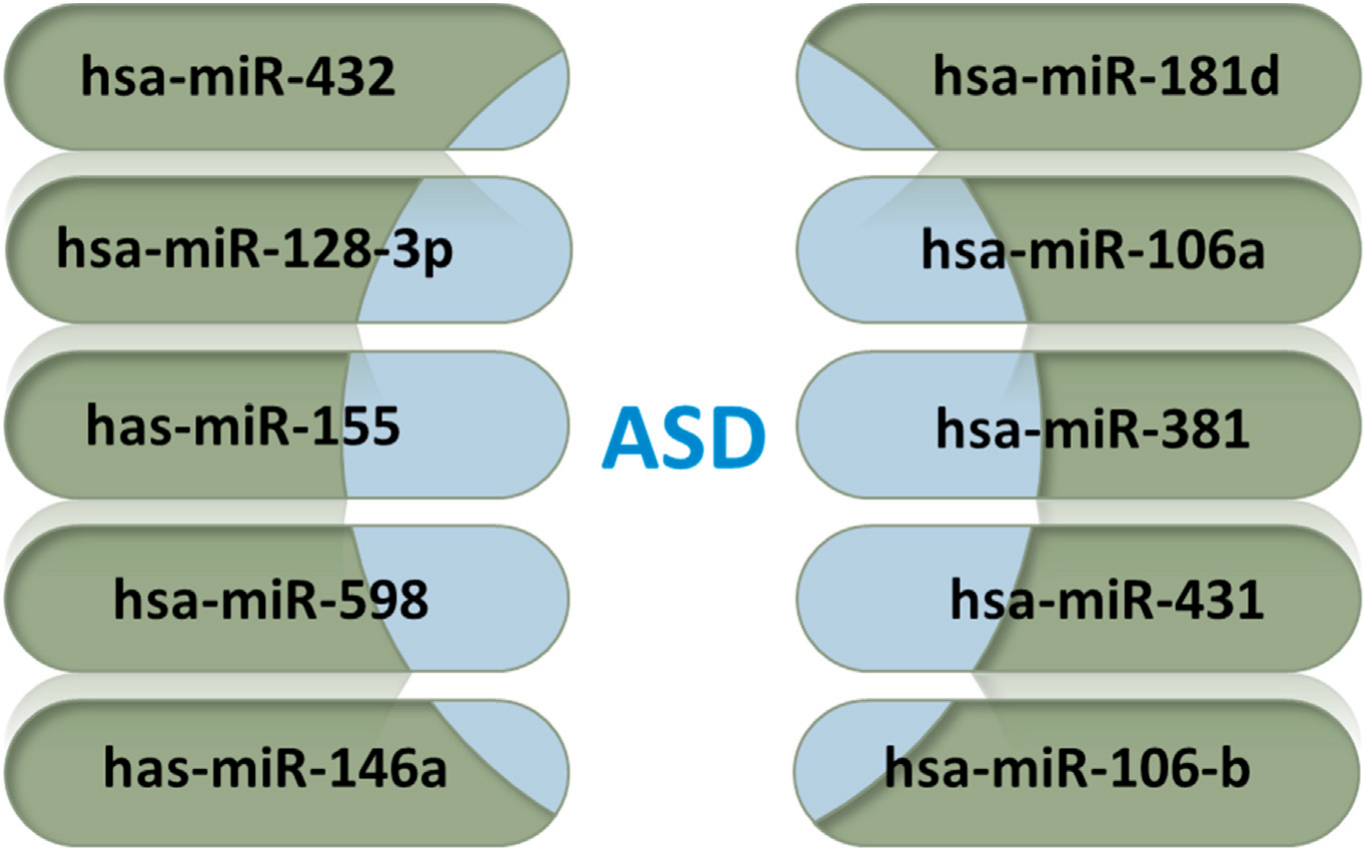
miRNAs associated with ASD as retrieved from mirNet database.

miRNAs can be detected in saliva, serum, plasma or blood as they are relatively stable. This makes miRNAs a reliable diagnostic tool. Recent studies indicated the potential use of miRNA as a biomarker for ASD. A study led by Hicks (2016) [121] concluded that miR-486-3p and miR-557 expression levels were increased significantly in autistic patients compared with the controls. Yu *et al.* [122], also demonstrated that miR-557 and miR486-3p expression levels were significantly increased (*p* < 0.05) in 18 autistic individuals compared with 20 controls. mirNet database searching revealed eight specific miRNAs related to ASD (Figure 2), and their target genes were also identified using microrna.org database (Table 2). A study found that miR-146a and miR-155 are overlapping miRNAs that are dysregulated in both ASD and atopic dermatitis subjects, thus suggesting a common etiology that could affect both neurodevelopmental and immune conditions [123].

**Table 2:** ASD-associated miRNA and their target genes in two databases.

miRNA	Target genes	Database
hsa-miR-598	2,191	In microrna.org (http://www.microrna.org)
hsa-miR-432	7,140	In microrna.org (http://www.microrna.org)
hsa-miR-128-3p	0	529 in mirNet (https://www.mirnet.ca)
hsa-miR-181d	8,084	In microrna.org (http://www.microrna.org)
hsa-miR-431	6,133	In microrna.org (http://www.microrna.org)
hsa-miR-106-b	0	0 in mirNet (https://www.mirnet.ca)
hsa-miR-381	8,544	In microrna.org (http://www.microrna.org)
hsa-miR-106a	9,121	In microrna.org (http://www.microrna.org)
Has-miR-146a	6,798	In microrna.org (http://www.microrna.org)
Has-miR-155	5,445	In microrna.org (http://www.microrna.org)

## 5 Conclusion

The role for DNA methylation in autism spectrum disorder has been supported by evidence from different research studies. The mechanism of such epigenetic changes is still mysterious and likely to be heterogeneous in nature, it could be due to changes in transcription levels of critical gene products during certain stages of brain development, caused by genetic and/or environmental factors. Further studies in identification of loci of promoters, miRNA or other genetic signatures affected by such epigenetic changes would help in better prevention of the recent epidemics of autism spectrum disorder. Furthermore, because of limited number of reports on the actual specific microbial taxon in guts of ASD patients, extensive research in this regard would be necessary.

## Declarations

### Author contribution statement

All authors listed have significantly contributed to the development and the writing of this article.

### Funding statement

This research did not receive any specific grant from funding agencies in the public, commercial, or not-for-profit sectors.

### Data availability statement

Data included in article/supplementary material/referenced in article.

### Declaration of interests statement

The authors declare no conflict of interest.

### Additional information

No additional information is available for this paper.
